# Supplementary material for: Comparative Molecular Characterization and Pharmacokinetics of IgG1-Fc and Engineered Fc Human Antibody Variants to Insulin-like Growth Factor 2 Receptor (IGF2R)
Source: Molecules. 2023 Aug 3;28(15):5839. doi: 10.3390/molecules28155839 (PMC10420659; doi:10.3390/molecules28155839)
Supplement: Supplementary file 1 [file molecules-28-05839-s001.zip › molecules-2516231-supplementary.pdf]

Supplementary Material:

a

b

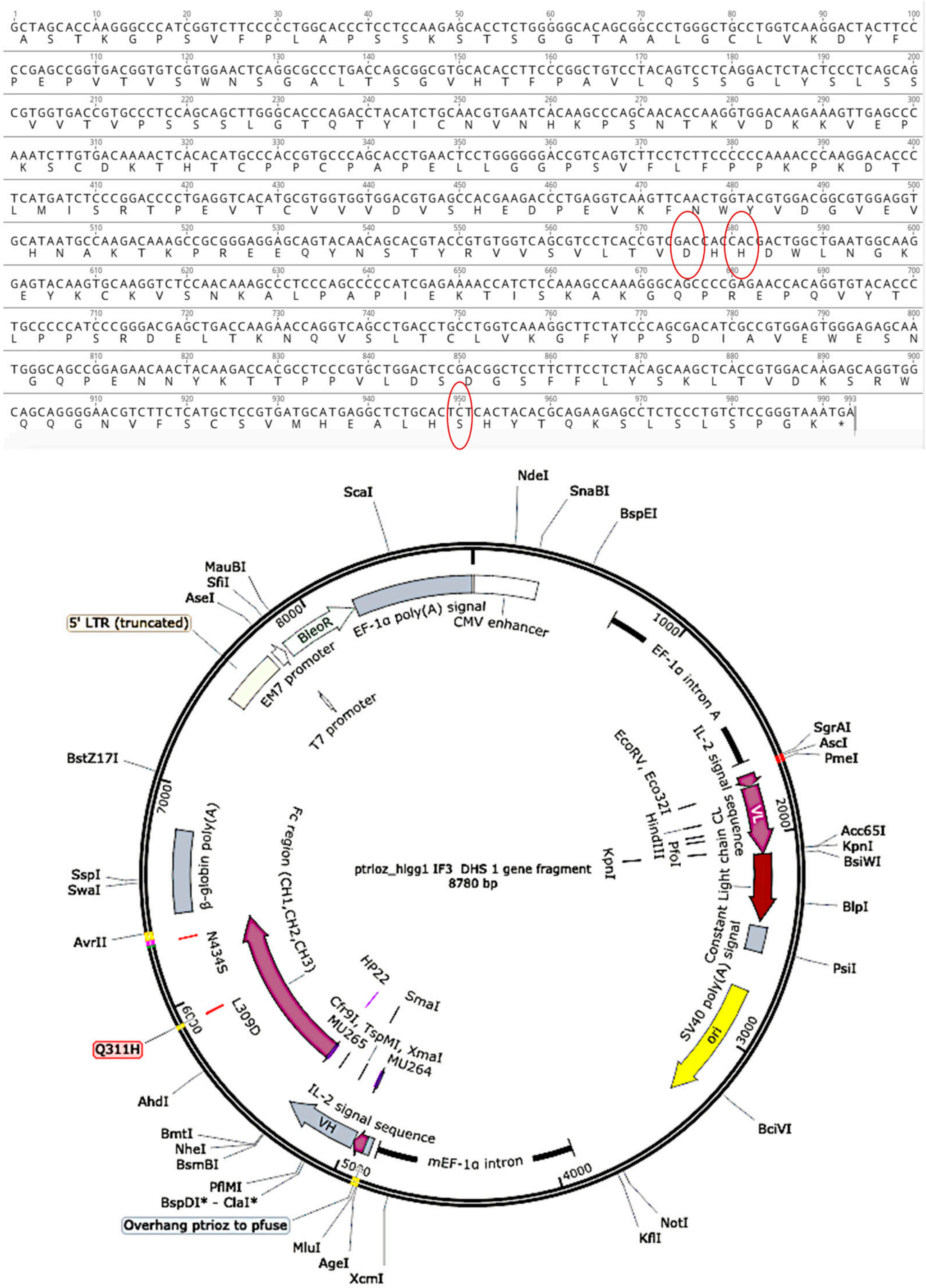

Figure S1 (a) Sequence of the cloned Fc region highlighting L309D Q311H and N434S substitutions and (b) pTRIOZ-hIgG1 (bicistronic) vector map that includes VL, VH and antibiotic selection cassette.

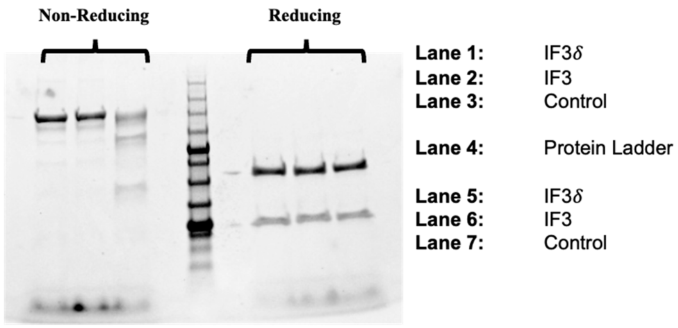

Figure S2 SDS-PAGE analysis of purified IgG1(IF3 and IF3δ) using Mab-select-sure affinity column. The gel electrophoresis was carried out in both reducing and non-reducing conditions.

a

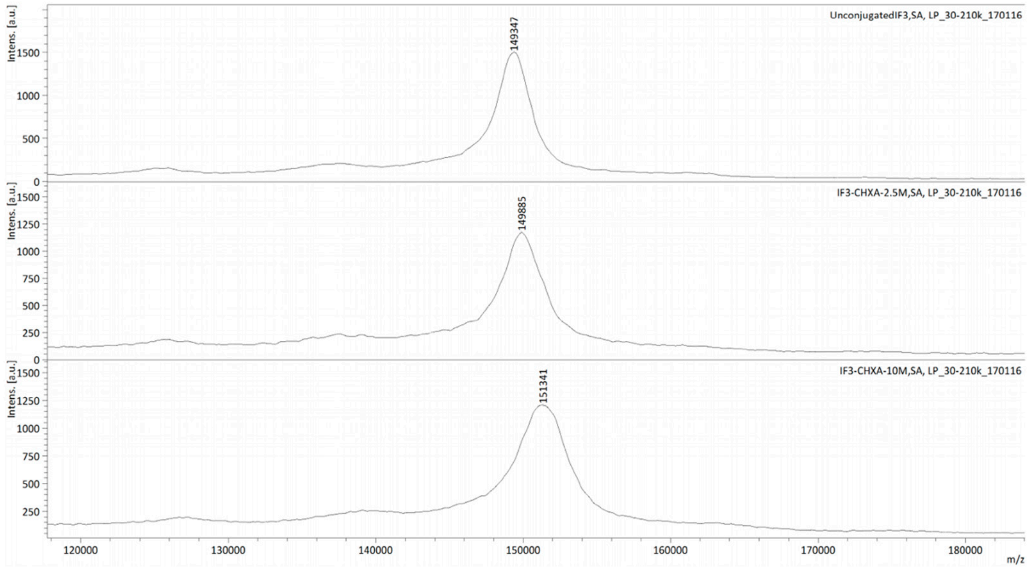

b

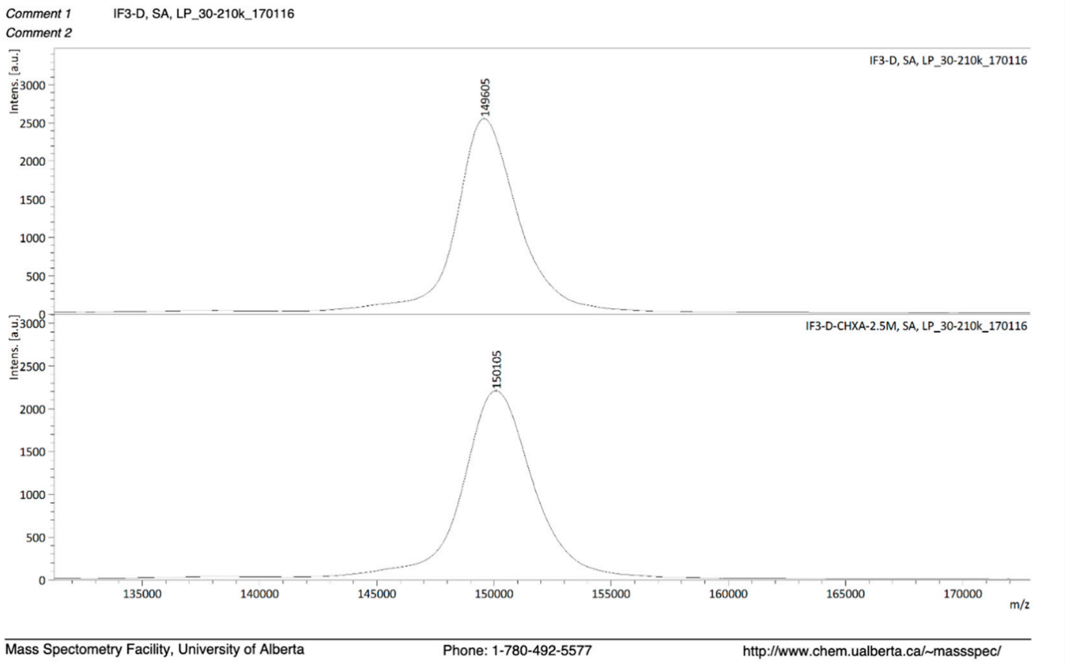

Figure S3 Determination of conjugated antibody ratio (CAR) for CHXA″-DTPA-IF3 and IF3δ using MALDI-TOF. The (CAR) are follows (a) CHXA″-DTPA/IF3 IgG1-The upper trace is unconjugated IF3, middle is 2.5M CHXA″-DTPA/IF3 IgG1, lower trace 10M CHXA″-DTPA/IF3 IgG1 and (b) CHXA″-DTPA/ IF3δ IgG1-The upper trace is uncojugated IF3δ, lower trace is 2.5 M CHXA″-DTPA/ IF3δ IgG1

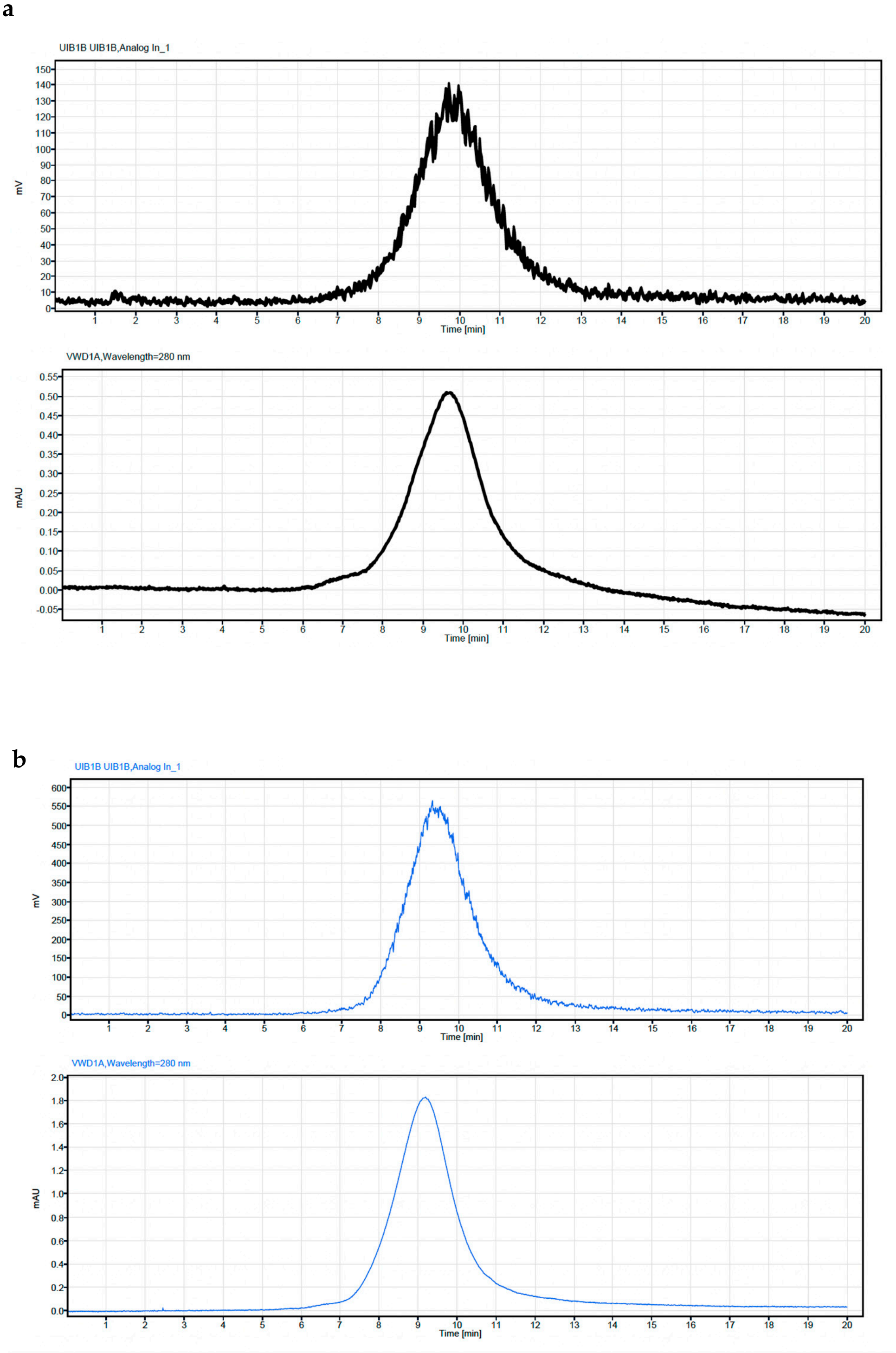

Figure S4 radioHPLC of ^{111}In-labeled antibodies. of radiolabeled (a) IF3 and (b) IF3δ showing both radiotracer and absorbance peak. Upper trace on both (a) and (b) is radioactivity, lower trave – UV absorbance at 280 nm.
